# CORE: A Phylogenetically-Curated 16S rDNA Database of the Core Oral Microbiome

**DOI:** 10.1371/journal.pone.0019051

**Published:** 2011-04-22

**Authors:** Ann L. Griffen, Clifford J. Beall, Noah D. Firestone, Erin L. Gross, James M. DiFranco, Jori H. Hardman, Bastienne Vriesendorp, Russell A. Faust, Daniel A. Janies, Eugene J. Leys

**Affiliations:** 1 Division of Pediatric Dentistry, College of Dentistry, The Ohio State University, Columbus, Ohio, United States of America; 2 Division of Oral Biology, College of Dentistry, The Ohio State University, Columbus, Ohio, United States of America; 3 Department of Biomedical Informatics, College of Medicine, The Ohio State University, Columbus, Ohio, United States of America; J. Craig Venter Institute, United States of America

## Abstract

Comparing bacterial 16S rDNA sequences to GenBank and other large public databases via BLAST often provides results of little use for identification and taxonomic assignment of the organisms of interest. The human microbiome, and in particular the oral microbiome, includes many taxa, and accurate identification of sequence data is essential for studies of these communities. For this purpose, a phylogenetically curated 16S rDNA database of the core oral microbiome, CORE, was developed. The goal was to include a comprehensive and minimally redundant representation of the bacteria that regularly reside in the human oral cavity with computationally robust classification at the level of species and genus. Clades of cultivated and uncultivated taxa were formed based on sequence analyses using multiple criteria, including maximum-likelihood-based topology and bootstrap support, genetic distance, and previous naming. A number of classification inconsistencies for previously named species, especially at the level of genus, were resolved. The performance of the CORE database for identifying clinical sequences was compared to that of three publicly available databases, GenBank nr/nt, RDP and HOMD, using a set of sequencing reads that had not been used in creation of the database. CORE offered improved performance compared to other public databases for identification of human oral bacterial 16S sequences by a number of criteria. In addition, the CORE database and phylogenetic tree provide a framework for measures of community divergence, and the focused size of the database offers advantages of efficiency for BLAST searching of large datasets. The CORE database is available as a searchable interface and for download at http://microbiome.osu.edu.

## Introduction

Large datasets consisting of hundreds of thousands and even millions of sequences are produced with high-throughput sequencing technologies, and developing methods for accurate and efficient analysis of these datasets is a growing challenge. It is currently computationally intractable to make individual taxonomic assignments with *de novo* phylogenetic tree construction approaches for such large numbers of sequences. In order to make taxonomic divisions for large 16S rRNA gene datasets two fundamentally different approaches have been used. 16S rDNA sequences from bacteria have been grouped into operational taxonomic units (OTUs) with distance-based agglomerative clustering approaches such as MOTHUR [1] Cd-hit [2], and QIIME [3]. Alternatively 16S rDNA sequences have been identified and classified by comparing novel sequences to a comprehensive reference database for which taxonomic assignments have previously been made. General reference databases include the GenBank nucleotide database (www.ncbi.nlm.nih.gov/nuccore) and the more highly curated and specialized Ribosomal Database Project (RDP) (rdp.cme.msu.edu/), SILVA (www.arb-silva.de) and greengenes (greengenes.lbl.gov) databases. Tools for identification and assignment of sequences against databases include Basic Local Alignment Search Tool (BLAST) [4], BLAST-Like Alignment Tool (BLAT) [5], RDP Sequence Match [6] and the RDP Classifier [7].

Agglomerative approaches such as MOTHUR [1] are particularly valuable for characterization of understudied microbial communities because they can be applied without prior knowledge of the taxonomy of the community. But a disadvantage for analysis of better-defined communities is lack of connection to taxonomic assignments made on the basis of information beyond the 16S sequence. Although the general concept of species divisions for bacteria has been regarded as problematic, there are a number of species definitions that are meaningful in the context of human health and disease, and being able to use this information with 16S sequencing efforts is important. Another problem inherent in agglomerative approaches is the inability to link short sequences from different regions of the 16S gene that belong to the same OTU.

Identification of 16S sequences by comparison to curated databases offers the potential to address these problems. Taxonomic information can be attached to sequences, and fragments from different regions of the same 16S sequence can be linked. Short sequences can be matched to longer reference sequences for which it is possible to more robustly reconstruct phylogeny and make taxonomic assignments. However, despite curation efforts, many of the sequences in the large public databases are of questionable quality [8], [9], [10], [11], or are inaccurately classified. A study using a Bayesian classifier found that as many as 5.5% of near full length sequences from well-characterized species were taxonomically anomalous at the level of genus in a test against the RDP [7]. The problem of identification is undoubtedly greater for the shorter reads of high-throughput sequencing methods, for uncharacterized OTUs, and at the level of species, which may be critical for human microbiome studies.

In addition, the number of incompletely characterized sequences in the public databases has been growing at a rapid rate, especially in GenBank. As a result, when new 16S sequence data are compared via BLAST to GenBank and other public databases, the high-scoring pairs may include only uncultured and unclassified bacteria that are of little use for identification and taxonomic assignment. The oral microbiome includes many uncultivated and unclassified bacteria, making studies to elucidate the etiology of chronic oral infectious diseases, such as dental caries and periodontitis, challenging.

For the large datasets generated using high-throughput methods it is impractical to manually examine and correct taxonomic assignments, so the development of accurate and efficient approaches to characterizing the human microbiome is essential. In order to address these problems, we developed a 16S database of the core human oral microbiome (CORE). The intent was to include a comprehensive, minimally redundant representation of the bacteria that regularly reside in the human oral cavity with computationally robust classification at the level of species and genus. The oral microbiome of humans has been studied extensively, and nearly half of the species-level taxa detectable by 16S rDNA analysis have been cultured, characterized and named. This provided the starting material for assembly and curation of the database. The database was expanded using clinically derived 16S sequences from a large number of subjects, by deep sequencing of samples, and extensive curation using computational methods. A number of classification and naming inconsistencies, especially at the level of genus, were resolved, and species and genus-level groupings were made for sequences from uncultivated taxa. This resulted in a database that performed much better than large public databases for identification of human oral 16S sequence data by several criteria. In addition, the resulting phylogenetic tree provides a framework for measures of divergence such as UNIFRAC [12] that are useful for the comparison of health and disease-associated communities. The development and testing of the CORE database is described in the following sections. The CORE database is available as a searchable interface and for download at microbiome.osu.edu.

## Methods

### Initial sequence selection

An initial list was generated by extracting the names of taxa (species and phylotypes) from published surveys of oral bacterial communities conducted using 16S rDNA sequencing [13], [14], [15], [16], [17], [18], [19], [20], [21]. GenBank was then searched for 16S sequences from each of the taxa. The initial list included 3131 sequences. Highly similar sequences were grouped using a script coded in PHP, and one representative was manually selected from each group. A 0.3% similarity threshold was empirically chosen as a level of divergence that would allow for sequence errors that might occur between identical sequences, about 5 nucleotide positions in the approximately 1.5 kb 16S sequence. The script took the first sequence as a query for BLAST against the remaining unmatched sequences. The script then formed a group based on the query sequence plus those sequences within the 99.7% similarity threshold. A sequence was then randomly selected from among the remaining, unmatched sequences and the BLAST process repeated. This was done until no ungrouped sequences remained. The criteria for selection of a group representative included length and quality of sequence as well as the quality of the metadata for the sequence (i.e. strain deposited in public database, type strain for a species, human and oral provenance). The large amount of publicly available sequence often includes genetic variability within species, and multiple sequences within a taxon were retained if divergence was >1%. Since the database is designed to find sequences that are 98% identical by BLAST, multiple highly similar sequences do not need to be retained. After this step the database consisted of 1235 sequences. A single representative from each bacterial phylum that was not otherwise represented was added to facilitate identification of novel clinical sequences.

### Phylogenetic analysis

Once sequences were collected in the database, multiple sequence alignments were generated with ClustalW [22]. Alignments were generated for the entire database and separately for each phylum. In the case of the Firmicutes and Proteobacteria, which represent a large number of species, alignments were generated for each class. Alignments were examined in Mesquite [23], manually edited, and the 5′ and 3′ ends were trimmed to minimize areas with missing sequence. The edited and trimmed multiple sequence alignments were used as the input for phylogenetic analysis. For the whole database alignment maximum likelihood trees were calculated using the MPI version of raxmlHPC [24] on a Linux cluster of 32 PCs containing 32 AMD Opteron cpus. For single phylum alignments the web server RAxML BlackBox [25] was used. Analyses were performed selecting the tree with the best log-likelihood out of a set of 1000 replicates, using the GTRGAMMA model of nucleotide substitution. The model of substitution was based on the suggestion by MODELTEST (http://darwin.uvigo.es/software/modeltest.html). Trees were viewed using Dendroscope [26] or ITOL [27]. Sequences on long branches or with unusual topology were critically reexamined. Sequences with 10% or more undetermined bases or chimeric features, as determined by screening with MALLARD [10] followed by manual examination of alignments generated by BLAST against GenBank, were removed.

### Assigning Supported Operational Taxonomic Units (OTU)

The sequences were divided into OTUs based on multiple criteria including maximum-likelihood-based topology and bootstrap support, genetic distance, and previous naming. The first step was to examine maximum-likelihood-based topology. Bootstrap values of 70% have been previously estimated to correspond to 95% confidence [28], so a bootstrap value of ≥70% was considered to be statistical support for the separation of sister groups. Evolutionary distance was then considered. We have used the NCBI taxonomy where possible in naming.

#### Species-level OTUs

Nearly half the taxa comprising the oral microbiome have been characterized and named. These names were maintained wherever possible. Prior to assigning species level OTU designations, sequence groupings were generated with similarity thresholds of 97, 98 and 99 percent using MOTHUR [1] and examined. Ninety-eight percent corresponded most closely with previously established species divisions, and therefore was adopted as the threshold for species-level OTUs for the oral microbiome.

A nearest neighbor clustering approach was used whereby sequences were included in a species-level OTU if they were ≥98% similar to any sequence within that cluster. There were instances when all three criteria (previous naming, evolutionary distance, and bootstrap support) could not be met. In these cases distance was the deciding factor.

#### Genus-level OTUs

Clades were next examined at the level of genus using the same criteria described previously, except that a uniform distance threshold was not applied since it would have resulted in massive changes in previous genus designations. Instead previous naming at the level of genus was maintained wherever possible, and unnamed sequences were assigned to named genera when doing so resulted in a monophyletic group. In some cases no named genus could be found that represented a group of uncultured organisms. These clades were labelled as “unclassified” followed by the most specific taxonomic level that could be given using the NCBI taxonomy scheme.

### Iterative database curation

The database was refined in a series of steps, using newly acquired sequence data at each stage. In the first stage we used 20,000 16S sequences generated by Sanger sequencing by our research group from human dental plaque and pharyngeal samples from 200 different human subjects. In the second stage we used 1,304,223 sequences generated by FLX amplicon pyrosequencing from the V4 region of the 16S gene from 75 patients, while the third stage utilized 523,359 sequences from the V1 region from a 60-patient subset of the V4 group. In each case, we searched the database with BLAST and assigned sequences to their best matches in the database if they were at least 98% similar. We grouped the remaining sequences using the in-house written PHP script described above. For groups containing more than 25 sequences, we selected a representative and performed a BLAST search against the GenBank nucleotide database. If sequences longer than our test sequence matched at greater than 98% sequence similarity, we added that sequence to the database. If multiple sequences were found, we used the following criteria to select one: (1) greatest length (2) highest level of identity (3) oral cavity or airway provenance. After adding sequences we repeated alignments, chimera checking, tree building, and taxonomic assignment. For a few groups, we found no sequences with 98% or greater identity in public databases. For these groups, we inspected an alignment of the group, selected a representative member, and included that in the database. These sequences have been deposited in GenBank (accession numbers HM358597-HM358635).

Finally we re-ran the BLAST search of all clinically derived sequences against the CORE database and filtered out those CORE sequences for which fewer than 25 clinically derived sequences matched at >98% identity. This was done to minimize inclusion of sequences with artifacts such as chimeras or errors. It was also used to exclude contaminating environmental bacteria that are not residents of the oral cavity but might be present at low levels.

### Variation map

To determine the sequence variation along the 16S gene for the oral microbiome dataset, sequences were aligned using ClustalW [22], and variation in base composition at each position was computed in Mesquite [23]. Gap characters were not included. The Shannon entropy index (H') [29] was calculated for each base position as: 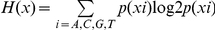, where p(x_i_) represents the probability of each base i at position x. An average entropy value was calculated with a variable window size, sliding at 1 bp intervals.

### Website construction

The website for microbiome.osu.edu was programmed using the Ruby on Rails framework (rubyonrails.org). Database searches are performed in the background using NCBI's blastn program [4]. The JAVA application Archaeopteryx (phylosoft.org/archaeopteryx), the successor to ATV [30], is used for the visualization of annotated phylogenetic trees. The application runs on an 8-CORE x86-64 Linux server with an nginx webserver (wiki.nginx.org) and MySQL (dev.mysql.com/) database.

### Generation of a test dataset and BLAST searches

The performance of the CORE database for identifying clinical sequences was compared to that of 3 other publicly available databases using a set of pyrosequencing 16S amplicon sequencing reads not generated by our research group and not used in the curation of the CORE database. The use of these novel sequences as a test allows comparison of the different databases to each other in an unbiased manner. The test sequences were downloaded from the HMP-DACC (http://www.hmpdacc.org/) and sequences were extracted from files containing subgingival data representing 24 patients, four sequencing centers and two regions of the 16S gene. Sequences were trimmed to remove primers and filtered to be over 350 bp in length and over 25 in cumulative quality score. A total of 1714 sequences were checked with Chimera Slayer (http://microbiomeutil.sourceforge.net/) and 87 apparent chimeras were discarded. Mallard could not be used for this purpose as it requires full-length 16S sequences. Following chimera removal, sequences were randomly discarded until 500 reads corresponding to each of the two 16S regions remained (V1–V3 and V3–V5). These 1000 sequences were used as queries for BLAST searches. The following databases were accessed: (1) NCBI GenBank Nucleotide Collection (ftp://ftp.ncbi.nih.gov/blast/db/, accessed 06/02/2010) (2) The Ribosomal Database Project (RDP release 10.20) (http://rdp.cme.msu.edu/, accessed 02/09/2009) (3) The Human Oral Microbiome Database 16S rDNA RefSeq Version 10.1 (HOMD) (http://www.HOMD.org/, accessed 05/29/2010), and CORE (version 5/29/10). The HOMD [31] includes a publicly available database of curated 16S rDNA sequences from the human oral cavity. NCBI BLAST version 2.2.23 (standalone) was used to search the 1000 sequence reads against each database. The following parameters for blastn were used: -task megablast –gapopen 0 –gapextend 0 –penalty -2 –reward 1 –dust no. All three performance tests (sequence identification, completeness, and ambiguity) used this 1000 sequence test set as BLAST queries. In each case the results were sorted on percent identity rather than e-value since the latter value can be biased by random differences in the length of database sequences.

### Leave one out cross validation analysis

We did BLAST searches with each of the database sequences against the complete database, using the same parameters as above. We ignored the exact match of each query against itself and identified the second best match. This was defined as the subject sequence with highest percentage identity under the additional criterion that the aligned region had to be at least 80% of the length of the shorter of the query and subject. The reason for using percent identity is discussed above, while the second criteria was needed to exclude occasional BLAST results that had high identity but only for very short aligned regions. We then filtered the second hits to queries from genera that contain more than one database sequence, and asked whether the genus-level OTU that we attributed to the query was the same as the one for the subject, counting those as successful classifications at genus level.

## Results

### Summary of database composition

**Figure 1:**
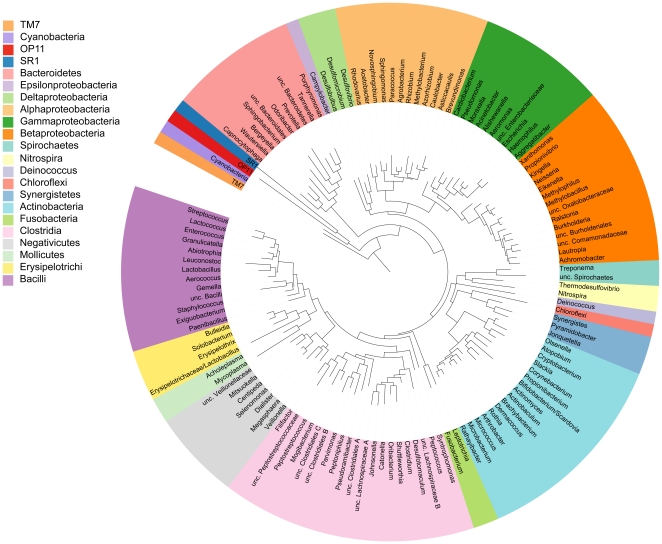
Circular phylogenetic tree at level of genus. The tree was generated with RAxML and viewed in ITOL [27]. Genera are color-coded by phyla, except for the Firmicutes and Proteobacteria, which are shown at the level of class.

**Figure 2:**
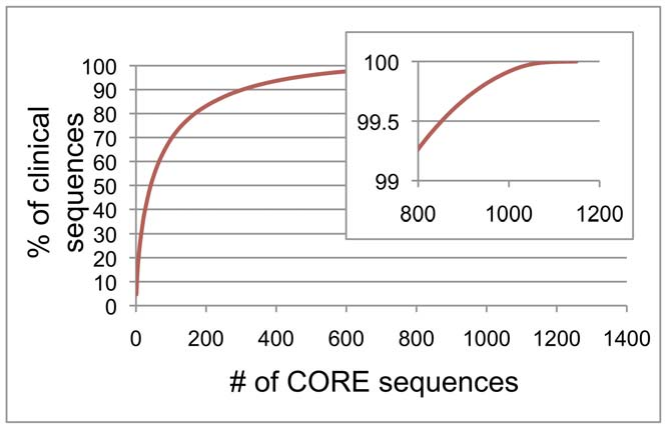
Cumulative distribution of clinical sequences against database entries. The frequency with which each of the sequences in CORE were encountered in the clinical datasets used for curation are shown as the cumulative percent of total sequences. They are ordered from most to least common. The majority of clinical sequences were accounted for by fewer than 1000 CORE entries.

**Figure 3:**
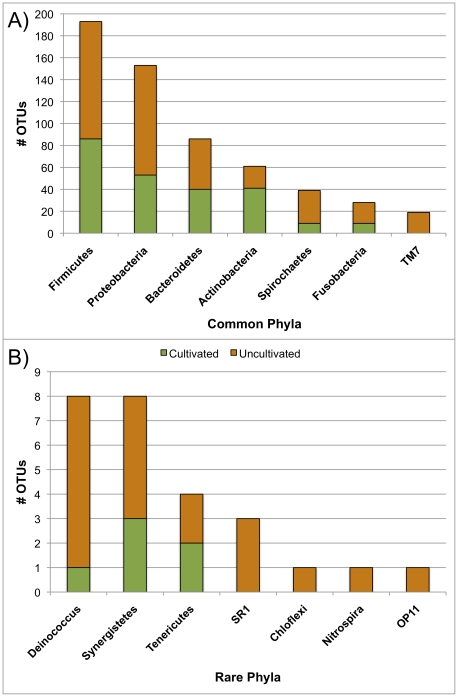
Numbers of S-OTUs by phylum in CORE. Number of S-OTUs assigned to each of the 14 phyla observed in the oral cavity and pharynx. A) Common phyla B) Rare phyla (<10 S-OTUs). The fraction of S-OTUs for which a cultivated member has not been reported is indicated.

Summary statistics for the CORE database are given in Table 1. There are 18 unclassified genus-level clades in the current version of the database. A phylogenetic tree at the level of genus is shown in figure 1. Detailed phylogenetic trees are at microbiome.osu.edu. Figure 2 shows the cumulative distribution of clinical sequences blasted against the database. Fewer than 800 database entries accounted for more than 99% of the sequences from the clinical samples used to curate the database. Figure 3 shows the number of OTUs in the CORE database by phylum and indicates the fraction that are cultivated species. As far as we can determine, 365 of the 636 assigned S-OTUs do not have cultured members.

**Table 1:** CORE Database Statistics.

No. of sequences	1043
No. of species-level OTUs	636
No. of genus-level OTUs	152
Average divergence within species-level OTU	1.3% (SD 0.8%)
Average divergence within genus-level OTU	7.3% (SD 5.5%)

### Variation map

**Figure 4:**
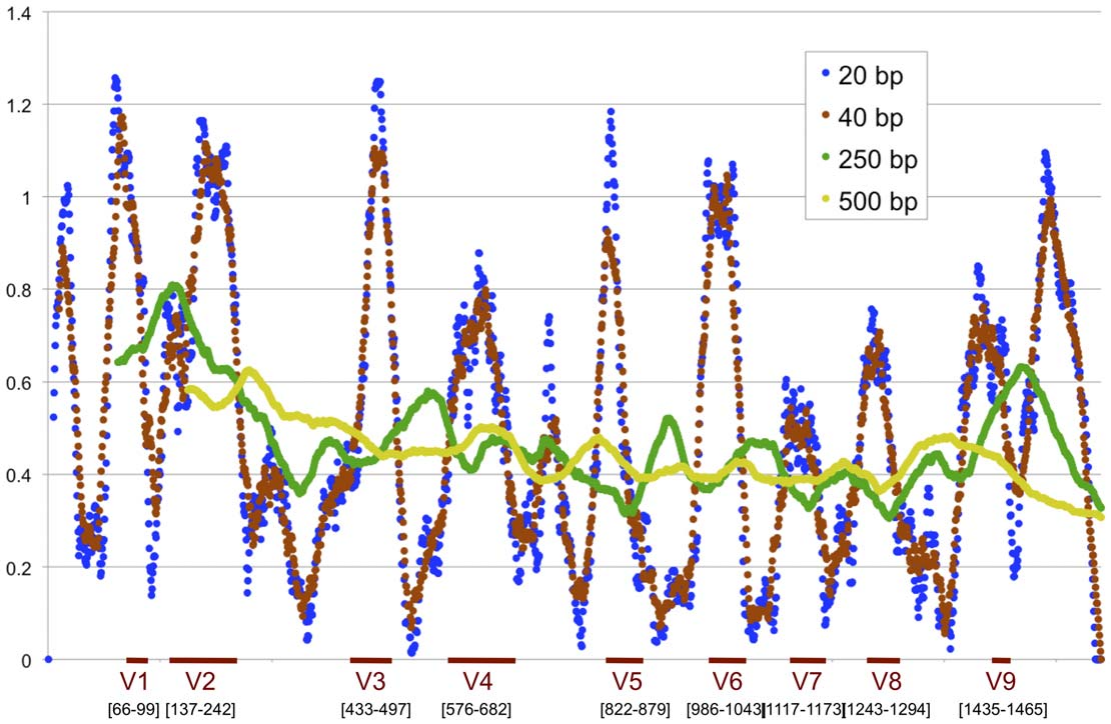
Plot of the variability of the 16S gene within the oral microbiome. 668 full-length 16S sequences selected to comprehensively represent the oral microbiome were aligned. The Shannon entropy index (H’) was calculated for each base position, and mean information entropy for primer-sized and amplicon-sized windows along the length of the sequence were plotted. Variable and conserved regions can be visualized. (Because of gaps inserted in the alignment the numbering does not correspond directly to *E. coli* numbering.)

Figure 4 illustrates the variation along the 16S sequence for primer-sized and amplicon-sized windows. The numbering of the aligned positions was adjusted to correspond to *E. coli* numbering [32], and the variable regions were mapped onto the graph.

### Comparison of performance among databases

#### Sequence identification

**Figure 5:**
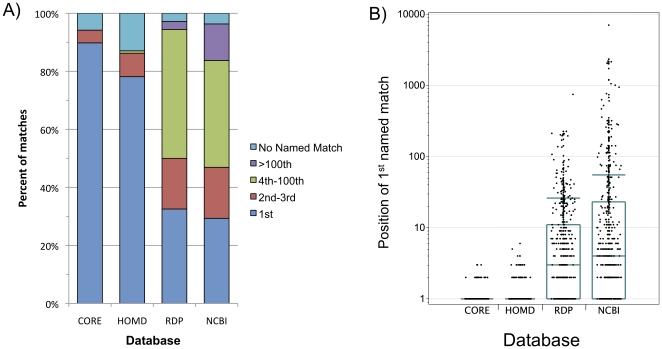
Position of 1st named match in BLAST results. A 1000 sequence test set of clinical sequences was BLAST searched against 4 databases. We ranked the results by sequence identity level (more appropriate than e-value because of the presence of truncated database sequences in some cases) and scanned the lists above the 98% similarity level to find the position of the 1^st^ match that included a full Latin name (genus plus species). A) Bar graph showing the results for queries for which a named match was found in at least one of the 4 databases. B) Box and whisker plots of position of 1^st^ named match for queries that returned a >98% identical named match for all databases. The lower limit, middle line, and upper limit of the blue box indicate the 25^th^, 50^th^ and 75^th^ percentiles of the data respectively. The whiskers are 1.5 times the inter-quartile distance, and jittered data points are shown. For CORE and HOMD, the boxes and whiskers are compressed at the 1 value because of the large number of named matches in the first result for these two databases.

The number of hits against unnamed species returned before encountering the first occurrence of a named species in a list of BLAST results is compared in Figure 5 for each of the four databases. 84.2% of the test sequences gave a named match in at least one of the databases and were included in the analysis.

#### Completeness

**Figure 6:**
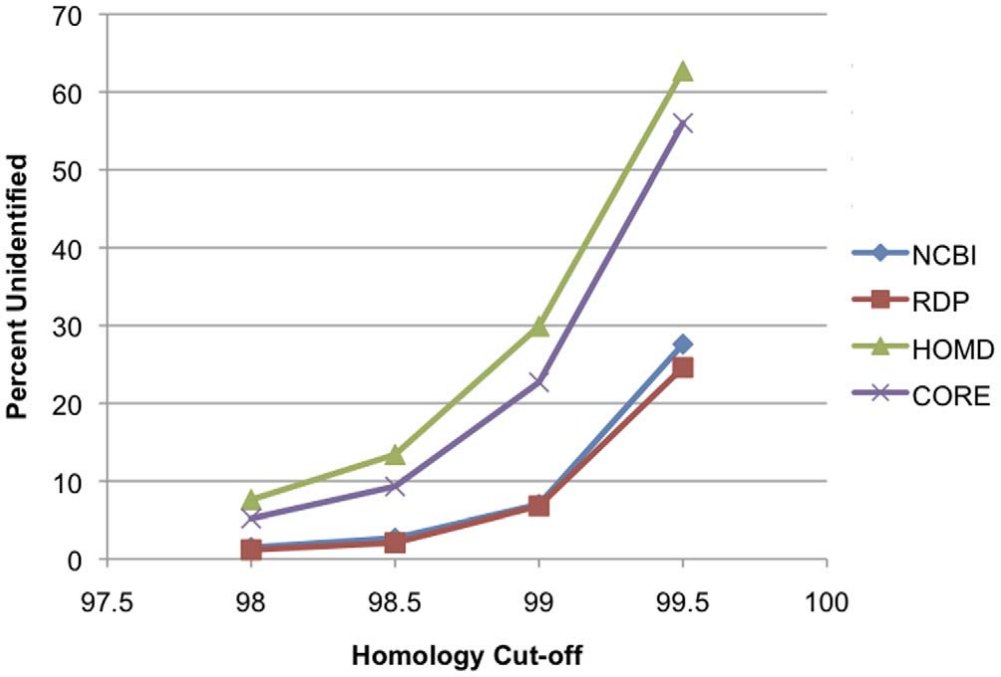
Completeness of databases. The percent of test sequences that failed to match any sequence is shown for each database for a range of similarity cut-offs.

As a measure of the completeness of the databases, the percentage of sequences that failed to match any sequence in each database using similarity score thresholds of 98%, 98.5%, 99%, and 99.5% is shown in figure 6. As expected the large databases were more complete and able to match previously unknown sequences at high percent identity. Due to its smaller size, CORE performed best at 98% identity, which is the threshold for which it was designed.

#### Ambiguity

**Figure 7:**
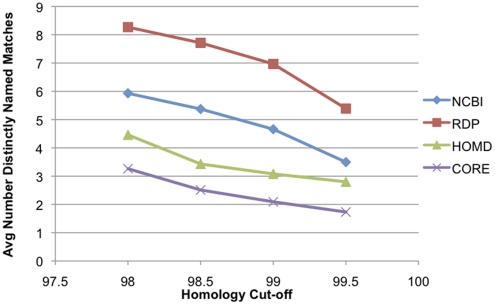
Ambiguity in databases. The mean number of species names that matched the test sequences is shown for each database for similarity thresholds from 98 to 99.5%.

Figure 7 shows the ambiguity in identification of the test set of 1000 sequences. The mean length of the test sequences was 489 bp. We performed a BLAST search with the 1000 test sequences against the 4 databases, then looked at all hits greater than the specified percentage identities, and counted how many different OTU names were present. The OTU designation was used as the basic taxonomic unit for CORE sequences, the GenBank organism field for NCBI, the oral taxon number for HOMD, and the sequence organism field for RDP.

### Leave one out cross validation analysis

We performed leave one out cross validation (LOOCV) analysis of the performance of CORE as described under Methods. The version of CORE that we used for this analysis contained 1043 sequences, and out of these 986 were from genera with more than one member. From those 986, there were 16 for which the second BLAST hit did not match at genus level, giving 98.4% successful classification. Of the misclassified queries, 9 out of the 16 came from genera that had only 2 or 3 sequences in the database. Out of the total database, only 78 sequences come from such genera, so they are clearly over represented in the misclassifications. We did not find any cases in which the same pair of sequences was misclassified with either as the query. We also performed this LOOCV analysis for the HOMD (http://www.HOMD.org/, accessed 03/10/2011) which gave 97% successful classification at the level of genus.

## Discussion

Designations have previously been made for over 250 cultivated OTUs from the oral microbiome, and we determined empirically that a similarity threshold of 98% for the 16S rDNA sequence most closely preserved these divisions. Using maximum-likelihood-based topology with bootstrap support and genetic distance for curation of an oral 16S database, another 365 uncultivated OTUs were defined, for a total of 636 computationally supported species level taxa. This is fewer than the 1,179 taxa reported by Dewhirst et al [33], who have collected a similar database. The difference between the two databases is the way in which taxa were defined, not in the range of sequence included. In fact, CORE tested as more comprehensive as discussed below. For the CORE database only computationally supported divisions were made, resulting in fewer divisions. The purpose of the CORE database is to provide the most accurate possible identification for unknown sequences. To this end phylogenetic analysis based on maximum likelihood and bootstrap support values was used to define taxa. It was clear that divisions based on previous species identifications or on distance analysis of 16S sequence in some cases are not supported, and in these cases taxa were combined to avoid ambiguous or erratic blast results.

The phylogenetic reconstruction of the oral microbiome did not indicate clear and unambiguous divisions based on a furthest neighbor clustering approach (whereby sequences are included in an OTU only if they meet a similarity threshold for *all* members of the cluster). So a nearest neighbor clustering approach was used whereby sequences were included in an OTU if they met the similarity threshold for *any* sequence within that cluster. Using this approach the average divergence within species-level OTUs was 1.3%, and the maximum distance was 4.6%. This approach did not lead to infinite expansion of the groups, suggesting that natural species-level divisions occur.

Some previously named, distinct species could not be distinguished from each other. An example occurred with the important group, *Streptococcus mitis, S. pneumoniae, S. infantis*, and *S. oralis*. There is not sufficient diversity in the 16S among these to establish them as independent taxa, therefore they were combined into a single clade and a combination name was used. In a few cases recognized species could be divided into subclades that were well supported and distant, and in these cases we divided the species into two clades. In a few cases sequences attributed to one named species appeared in well-supported clades represented by a different named species. In these cases, we assumed the discrepancy was the result of misidentification and renamed the sequence based on the supported clade.

The average similarity within previously named genus-level groups was 92.7%, somewhat less than the typically suggested 95% identity [34]. A large range was observed, with a similarity ≤ 85% within 10 genus groups. This unevenness along with many instances of ambiguity and inconsistency demonstrates that current naming is less than systematic. For example, the *Eubacterium* genus designation was particularly ambiguous, occurring broadly in unrelated clades. To provide nomenclature that is useful for epidemiologic studies corrected “genus group” designations based on previous naming and phylogenetic reconstruction were provided. These new genus groups are internally consistent as demonstrated by the LOOCV test, but the range is still large (78–97%). New categories based on a 95% similarity threshold would have so completely departed from previous naming that they were not attempted.

It was important to exclude from the database chimeras and other artifacts that could be misinterpreted to represent new taxa, and to exclude contaminating bacteria. The human oral cavity is an open system with exposure to a wide variety of environmental bacteria including soil, animal and plant bacteria present in food and water, so that an unfiltered survey would include many nonresident taxa. For this reason sequences that were not observed multiple times were removed from the database in curation.

To aid in the design of primers and amplicons for future sequencing efforts, we plotted the variation over the length of the 16S gene for the sequences contained in the database (figure 4). Results from primer and amplicon-sized windows shows the location of conserved regions as targets for primers and hypervariable regions as targets for amplicons.

### Database performance

#### Sequence identification

The most immediately obvious problem encountered in attempts to identify unknown sequences is the large amount of unidentified sequence in public databases. Both the GenBank nucleotide database and the RDP showed multiple instances in which hundreds and even thousands of sequences without taxonomic information appeared as the highest-scoring pairs in BLAST searches, even though there was a named species with 98% identity present in the database. CORE showed the best performance, but both HOMD and CORE always returned a named species within the 10 highest-scoring pairs if they contained a match, and the majority of the time it was the first hit.

#### Completeness

As expected, the large, general databases were the most complete. As can be seen, however, CORE performed well at 98% similarity score threshold, with 95% of the query sequences matching at least one database entry. The HOMD [31], another oral database, performed a bit less well, with 92% of the query sequences matching a database entry. The general databases provide an important supplement to the specialized databases for identification of rare sequences. In analyzing new datasets, our process is first to search CORE using BLAST, and unidentified sequences that occur with a high enough frequency are queried against the general databases for identification. They are added to the CORE database in an ongoing process of curation that increases the comprehensiveness of the database.

#### Ambiguity

Figure 7 shows that CORE produced the lowest ambiguity. The sequences used for this test were less than 500 bp in length, and longer sequences would show reduced ambiguity. The ambiguity encountered with CORE was almost entirely accounted for by sequences assigned to the Streptococci, which show low inter-species variation in the 16S and so are incompletely resolved with short sequences.

#### Leave one out cross validation analysis

LOOCV analysis of CORE shows it contains few taxonomic discrepancies, with a 98.4% accuracy at genus level. CORE compares favourably with both the HOMD [31] at 97% and the RDP classifier, with a reported 91.4% accuracy [7]. Most discrepancies in CORE were accounted for by a few rare taxa for which full length sequences were not available, and ongoing curation efforts will address this.

#### Overall utility

The better identification and phylogenetic classification of sequences achieved with the development of the CORE database has provided a substantially improved ability to separate health-associated communities from those associated with periodontitis (manuscript in preparation). This result is particularly striking at the level of genus where the standard taxonomy contained many ambiguities and errors. In addition, the resulting phylogenetic tree provides a framework for further comparative research in health and disease-associated bacterial communities with measures of community divergence such as UNIFRAC [12]. Database searches using BLAST to perform identification and classification of newly acquired sequences can be computationally intensive. This challenge has increased as high-throughput sequencing technologies increase the size of datasets. The availability of a targeted, well-curated database for oral bacterial 16S sequences provides a useful tool to minimize the time and resources needed for analysis.

#### Usage

The CORE database is available as a searchable web-based interface or for download at http://microbiome.osu.edu. It can be used either by submitting query sequences to a BLAST client at the above site, or by downloading the entire database and running command-line blast searches on a remote computer. It is anticipated that the second mode will be most useful to users with large datasets such as those from pyrosequencing. The relatively small size of CORE can speed searches of large numbers of queries relative to larger BLAST databases.

In addition to being used for BLAST searches to identify sequences, the CORE database could be used to train classifiers such as the RDP classifier [7] or the Bayesian classifier implemented in mothur [1]. Since discrepancies in classification have been minimized in CORE, it is likely that classifiers using CORE as a training set will show improved performance over less closely curated databases.

Specialized highly curated 16S rDNA databases outperformed larger public databases for the analysis of clinical datasets in important ways. Although the two larger databases, GenBank and RDP, returned named matches for a slightly higher fraction of the sequences, the focused databases, CORE and HOMD, were much more likely to accurately identify sequences. CORE provided the lowest level of ambiguity and was the more comprehensive of the specialized oral databases. The larger databases are still important supplements to the specialized databases for identification of rare sequences.
